# Risk Factors for Postoperative Bleeding after Adenoidectomy

**DOI:** 10.3390/children8030242

**Published:** 2021-03-21

**Authors:** Milan Urík, Michal Bartoš, Soňa Šikolová, Jana Jančíková, Klára Perceová, Jiří Jarkovský, Eva Klabusayová, Petr Štourač, Petr Jabandžiev

**Affiliations:** 1 Department of Paediatric Otorhinolaryngology, University Hospital Brno and Faculty of Medicine, Masaryk University, 61300 Brno, Czech Republic; urik.milan@fnbrno.cz (M.U.); bartos.michal@fnbrno.cz (M.B.); sikolova.sona@fnbrno.cz (S.Š.); jancikova.jana@fnbrno.cz (J.J.); perceova.klara@fnbrno.cz (K.P.); 2 Institute of Biostatistics and Analyses, Faculty of Medicine, Masaryk University, 61300 Brno, Czech Republic; jarkovsky@iba.muni.cz; 3 Department of Paediatric Anaesthesiology and Intensive Care Medicine, University Hospital Brno and Faculty of Medicine, Masaryk University, 61300, Brno, Czech Republic; klabusayova.eva@fnbrno.cz (E.K.); stourac.petr@fnbrno.cz (P.Š.); 4 Department of Pediatrics, University Hospital Brno and Faculty of Medicine, Masaryk University, Černopolní 9, 61300 Brno, Czech Republic; 5 Central European Institute of Technology, Masaryk University, 61300 Brno, Czech Republic

**Keywords:** bleeding, adenoidectomy, children, surgery, risk factors

## Abstract

IMPORTANCE: Postoperative bleeding is a common and potentially life-threatening complication. Precise identification of risk factors in addition to the basic ones, such as coagulation parameters, is certainly very desirable. OBJECTIVE: The aim of this study was to identify other possible risk factors for bleeding after adenoidectomy in children. DESIGN: This observational prospective study enrolled children undergoing adenoidectomy from October 2019 to February 2020, then evaluated the influence of possible risk factors for bleeding. SETTING: Tertiary pediatric otorhinolaryngology center. PARTICIPANTS: A total of 288 children aged 0–18 years undergoing adenoidectomy for recurrent upper respiratory tract infections, recurrent acute otitis media, secretory otitis media, and obstructive sleep apnea syndrome. MAIN OUTCOMES AND MEASURES: Increased blood pressure and time of surgery were identified as risk factors for bleeding after adenoidectomy. RESULTS: Elevated systolic (*p* = 0.046), diastolic (*p* = 0.012), and mean arterial blood pressure (*p* = 0.007) (Mann–Whitney U test) as adjusted for age-specific distributions and with corrections for height and weight, as well as time length of surgery (*p* < 0.001) (Fisher’s exact test) were revealed as statistically significant risk factors for postoperative bleeding. Atmospheric pressure, surgeon’s level of experiences, chronic inflammatory content in adenoid vegetation (AV), size of AV, recidivism of AV, recurrent infections of the upper respiratory tract, type of anesthesia, long-term using of drugs, and positive coagulation questionnaire or pathology in standard coagulation tests were not found to be risk factors for bleeding after adenoidectomy. CONCLUSIONS AND RELEVANCE: In this prospective study within a well-defined population of children, we evaluated increased blood pressure and time of surgery as risk factors for bleeding after adenoidectomy. These data bring new information that complements current knowledge in this field.

## 1. Introduction

Adenoidectomy (AT) is one of the most common surgeries in childhood. The main indications for AT include recurrent upper respiratory tract infections, recurrent acute otitis media, secretory otitis media, and obstructive sleep apnea syndrome. AT is a relatively safe procedure, but possibilities exist for complications. The most common and serious complication is postoperative bleeding, the incidence of which is 0.5–2.2% [1,2,3]. Many studies have evaluated different factors that cause bleeding after AT. These studies focus very often on the values of preoperative coagulation factors, as well as various types of surgical techniques [4,5,6]. In our prospective study, we focused on evaluating common factors (based upon an analysis of the literature) [7,8,9,10,11,12] and less common factors that may affect the risk of bleeding after AT. The identification of these risk factors could result in better care for these patients.

## 2. Methods

This was a prospective observational study from the Department of Pediatric Otorhinolaryngology (DPO), University Hospital Brno, Czech Republic. DPO is a pediatric tertiary center. Children aged 0 to 18 years were enrolled if they met the following inclusion criteria: (1) admission to the DPO, (2) indication of AT (due to recurrent upper respiratory tract infections, recurrent acute otitis media, secretory otitis media, and obstructive sleep apnea syndrome), and (3) signed informed consent by their parents or legal guardians. We excluded patients with a diagnosed/treated coagulation disorder from the study. The study was conducted from October 2019 to February 2020. Surgical approaches and anesthesiological procedures were the same in all patients and consisted of general anesthesia with orotracheal intubation, opening mouth with a spreader for AT, and nasopharyngeal curettage using an endoscope. Hemostasis was performed using tampons. In the case of prolonged bleeding, we used local hemostyptic drugs (Traumacel—carboxycellulosum calcium or Sanorin—naphasolini nitras 0.05%) or intravenous drugs (Exacyl—acidum tranexamicum) and electrocauterization of vessels. The experienced surgeon filled out a study questionnaire for each patient. The questionnaire contained patient identification data, age, sex, atmospheric air pressure (measured by official weather station), blood pressure (systolic, diastolic, and mean arterial pressure during surgery—mean value from three measurements during surgery), anesthesiological complications (difficult airway, desaturation, bradycardia, tachycardia, laryngospasm, and/or bronchospasm that might occur at induction of anesthesia or after extubation), adenoid vegetation (AV) size, AV content, recurrence of AV, a preoperative examination of coagulation parameters (all patients with coagulation parameters outside of the reference range were consulted with an experienced hematologist; if there was a need to administer any hematological treatment, then the patient was excluded from further analyses), experience of the surgeon (˂5 years or >5 years), time of surgery, and history of recurrent respiratory tract infections. Increased bleeding has been recorded if we used procedures other than tampons for hemostasis (hemostyptic drugs or cauterization). Only patients having complete questionnaires without missing data were enrolled into the study. 

The patients were divided into two groups as follows: Group A—with significant bleeding as defined above, and Group B—without significant bleeding.

The continuous variables were described using non-parametric statistics (median, minimum, maximum), as normality was not confirmed by Kolmogorov–Smirnov test, absolute and relative frequencies were used for categorical variables. To test for differences between groups A and B in values of continuous variables, Mann–Whitney U test was used. Fisher’s exact test was used to test for differences in distributions of categorical variables. Linear regression was used for age-adjusted comparison of MAP(Mean Arterial Pressure) between groups A and B. ROC(Receiver Operating Characteristic) analysis was used to find optimal cut-off values of continuous predictors (maximum sum of sensitivity and specificity). Logistic regression was used to find risk factors for post-operative bleeding. Significant predictors (*p* value < 0.05) were kept in the multivariate model (backward stepwise).

Age-specific distributions of systolic and diastolic blood pressure in infants and children were made, with corrections for height and weight [7].

This study was approved by the Institutional Ethical Committee of the University Hospital Brno in accordance with the 1964 Declaration of Helsinki.

## 3. Results

**Figure 1:**
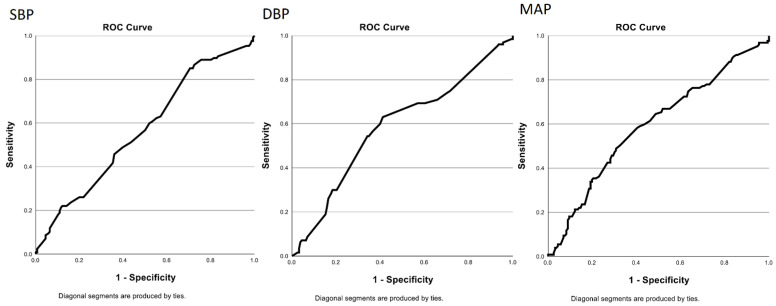
Univariate logistic regression. Maximum values of sum of sensitivity and specificity in ROC analysis.

The study enrolled 288 consecutive patients undergoing AT between 2 and 17 years of age. Median (min-max) age was 5 (2–16) years. Median age in both groups was 5 years. The mean systolic (SBP) and diastolic blood pressures (DBP) in group A were higher than in group B and this was statistically significant (Mann–Whitney U test, *p* = 0.046 and *p* = 0.012, respectively). The mean arterial pressure (MAP) was higher in group A than in group B, so MAP was a risk factor for bleeding (Mann–Whitney U test, *p* = 0.007). Seventy-four (25.7%) surgeries were performed in less than 15 min, 39 (13.5%) surgeries in more than 30 min. The time of surgery was associative with high risk of bleeding (Fisher’s exact test, *p* < 0.001). Before surgery, 36.8% of all patients had a coagulation questionnaire completed. Only in 2 of these patients were there risk events, and 1 of these 2 patients had prolonged bleeding during surgery. Before surgery, 237 patients (82.3%) had laboratory tests for coagulation performed. In 13 (4.5%), there were some pathology values, but we observed no risk of bleeding in these patients. In 140 patients (48.6%), there was chronic inflammation content in adenoid, but it was not a risk factor for bleeding. A surgeon with more than 5 years of experience performed 207 surgeries (71.9%) and 81 surgeries were performed by a skilled surgeon with less than 5 years of experience. The age and experience of the surgeon had no impact on bleeding after AT. In 12 (4.2%) patients, the indication for surgery was a recurrence of the adenoid tonsil. The recurrence was not a risk factor for bleeding. Anesthesiological complications (laryngospasm) occurred in only 11 (3.8%) patients, and these did not constitute a risk factor for bleeding. Recurrent upper respiratory tract infections characterized 247 patients (85.8%), and it was not a risk factor for bleeding. All findings are summarized in Table 1. A one-dimensional logistic regression model (with optimal cut-off value for pressure chosen using the ROC curve—Figure 1) and a linear regression model are depicted in Table 2 and Table 3, respectively. Using multidimensional logistic regression model, the age was not a risk factor for bleeding (*p* = 0.481), but MAP (*p* = 0.003) and time of surgery were risk factors again (*p* < 0.001), as shown in Table 4.

## 4. Discussion

This study marked the first time in a central European population of children that such a large cohort was evaluated in relation to less well-known risk factors for bleeding after adenoidectomy. 

It is known that the level of blood pressure significantly affects the risk of any surgical bleeding [13,14], so we monitored systolic, diastolic, and mean arterial blood pressure during the surgery. We showed that higher SBP, DBP, and MAP are associated with higher risk of bleeding. Age-specific distributions of systolic and diastolic BP in infants and children, with corrections for height and weight, are widely known [7] The mean SBP, DBP, and MAP naturally increase during childhood. With each year of age, the MAP increases by 0.71 mmHg. Even though the risk of blood pressure increase is not too high in absolute terms, it is statistically significant and may have important clinical consequences. In particular, this relates to the course of the preoperative period and that of anaesthesia itself. 

Type of general anaesthesia induction and maintenance, together with providing an adequate level of analgesia, has a significant impact on hemodynamic stability of the patient. Elevation of systolic blood pressure perioperatively is associated with postoperative complications, such as bleeding [15]. Many studies describe comparing inhalation anaesthesia induction with sevoflurane and intravenous anaesthesia induction with propofol. While sevoflurane is currently used in the vast majority of pediatric general anaesthesia cases, it has been shown that the inhalation induction and maintenance of general anaesthesia with sevoflurane significantly increases MAP [16]. On the contrary, inhalation induction is associated with better blood pressure maintenance compared to propofol [17]. Intravenous anaesthetics (especially in high dosing) and also increased doses of volatile anaesthetics are associated with decrease in blood pressure [17]. To prevent administering excessive doses of anaesthetics and therefore hemodynamic disturbances, the use of bispectral index monitoring to assess the depth of anaesthesia might be helpful [18].

Before adenoidectomy, patients must have blood tests with basic coagulation parameters or a completed coagulation questionnaire approved by the Czech Society of Haematology, Czech Pediatric Society, and Czech Society of Otorhinolaryngology and Head and Neck Surgery [19]. We found that the risk of bleeding is not higher in a patient with a positive coagulation test (some pathological values) or with a positive coagulation questionnaire. Many studies consider the coagulation questionnaire to be sufficient, effective, and more cost-effective than routinely performing laboratory coagulation tests [20,21,22].

Some studies show that the risk of bleeding and revision after AT is greater if indications for AT are obstructive sleep disorders (sleep apnea) or associated with middle ear disease (OME—otitis media with effusion) than if for recurrent respiratory infections.^8^ Our data show that the indication for AT is not a risk factor for bleeding. 

Surgeon experience and skills are often discussed in the literature as constituting a factor that may play a crucial role [23], but we observed this factor not to be associated with the risk of bleeding. The risk of bleeding was the same for the surgeons with experience >5 years and ˂5 years. 

It has been hypothesized that there might be a greater risk of bleeding in patients who undergo AT repeatedly for recurrence, but we proved that the recurrence of adenoid vegetation is not a risk factor for bleeding.

Most surgeries were completed within 15 min after curettage. Time of surgery longer than 30 min was associated with a higher risk of bleeding and revision surgery, but the fact is, of course, that time of surgery is prolonged where there is prolonged bleeding. In these cases, it is necessary to attend to the blood losses caused by repeated aspiration, drying with tampons, and replenishment of the circulating blood volume.

Many complications can potentially arise during anaesthesia. We found none of these complications to be common in our cohort, however, and we also did not observe them to have effect upon the risk of bleeding after AT. We also found long-term medication in patients not to be a risk factor for bleeding.

Patient age is one of the factors for bleeding, but the data are not conclusive. While some studies confirm that age is not a risk factor for bleeding [2], other authors have shown that the bleeding is very rare in children under 3 years [24] and that older age is associated with a greater risk of bleeding and need for revision in the operating room [1]. In our study, we did not show an effect of age on bleeding. Of course, as noted above, blood pressure increases with age, and that, according to some studies is a likely cause of more frequent bleeding in older children.

From the clinical point of view, it seems that in certain periods, a higher frequency of postoperative bleeding is observed. In an effort to reveal possible influences and in accordance with the literature data, we investigated the possible connection with changes in atmospheric pressure [25,26]. Although it turned out that the mean atmospheric air pressure associated with the group of children with bleeding was lower than that for the group without bleeding, the differences in this parameter were not statistically significant. There are no other studies in the literature wherein this factor was monitored, but we could hypothesize a possible influence of atmospheric pressure on blood pressure.

We see as a limitation of our study the total number of enrolled patients, which could have been higher and have covered a full 12-month year in the tertiary center. The worsening of an overall epidemiological situation (the COVID-19 pandemic) since March 2020 has forced us to end patient recruitment for the time being.

## 5. Conclusions

This is a unique prospective study that focused on the effect of risk factors for bleeding after adenoidectomy. We identified blood pressure as a risk factor for bleeding after adenoidectomy. One of the factors influencing blood pressure during adenoidectomy may be the type and management of anaesthesia. Possible risk factors often discussed in the literature, such as patient age, coagulation questionnaire, and common coagulation test, have not been shown to be significant. These findings could lead to improved care for these patients.

## Figures and Tables

**Table 1:** Characteristics of patients in analyzed groups.

		Total	Group A	Group B	
Factor	Value	N = 288	N = 159	N = 129	*p*-Value
Age (years)					0.118
Median		5	5	5	
Min-Max		2–16	2–14	2–16	
Atmospheric pressure (hPa)					0.142
Median		1005	1006	1004	
Min-Max		974–1031	977–1031	974–1031	
SBP (mmHg)					**0.046**
Median		105	105	106	
Min-Max		80–144	80–140	80–144	
DBP (mmHg)					**0.012**
Median		56	55	60	
Min-Max		40–90	43–90	40–90	
MAP (mmHg)					**0.007**
Median		73	72	74	
Min-Max		54–108	58–103	54–108	
Coagulation questionnaire	Negative	104 (36.1%)	58 (36.5%)	46 (35.7%)	0.952
	Positive	2 (0.7%)	1 (0.6%)	1 (0.8%)	
	None	182 (63.2%)	100 (62.9%)	82 (63.6%)	
AV size	˂1/3 of choana	49 (17.0%)	28 (17.6%)	21 (16.3%)	0.878
	˂1/2 of choana	115 (39.9%)	65 (40.9%)	50 (38.8%)	
	>1/2 of choana	124 (43.1%)	66 (41.5%)	58 (45.0%)	
Coagulation test	Negative	224 (77.8%)	123 (77.4%)	101 (78.3%)	0.354
	Positive	13 (4.5%)	5 (3.1%)	8 (6.2%)	
	None	51 (17.7%)	31 (19.5%)	20 (15.5%)	
AV content	No content	148 (51.4%)	80 (50.3%)	68 (52.7%)	0.723
	Chronic	140 (48.6%)	79 (49.7%)	61 (47.3%)	
Surgeon	>5 years’ experience	207 (71.9%)	107 (67.3%)	100 (77.5%)	0.065
	˂5 years’ experience	81 (28.1%)	52 (32.7%)	29 (22.5%)	
AV recidivism	No	276 (95.8%)	154 (96.9%)	122 (94.6%)	0.384
	Yes	12 (4.2%)	5 (3.1%)	7 (5.4%)	
Time of surgery	˂15 min	74 (25.7%)	54 (34.0%)	20 (15.5%)	**<0.001**
	15–30 min	175 (60.8%)	101 (63.5%)	74 (57.4%)	
	>30 min	39 (13.5%)	4 (2.5%)	35 (27.1%)	
Anesthesiology complications	No	277 (96.2%)	153 (96.2%)	124 (96.1%)	0.999
	Yes	11 (3.8%)	6 (3.8%)	5 (3.9%)	
URTIs	No	41 (14.2%)	18 (11.3%)	23 (17.8%)	0.129
	Yes	247 (85.8%)	141 (88.7%)	106 (82.2%)	

Abbreviations: hPa—hectoPascal unit, SBP—systolic blood pressure, DBP—diastolic blood pressure, MAP—mean arterial pressure, AV—adenoid vegetation, URTIs—upper respiratory tract infections. The bold means statistically significant results.

**Table 2:** Univariate logistic regression. Cut-off values were selected to obtain maximum values of sum of sensitivity and specificity in ROC analysis.

Predictor		OR (95% Confidence Interval)	*P*
Systolic blood pressure	≤99 mmHg	Reference	
	>99 mmHg	2.38 (1.31–4.32)	**0.004**
Diastolic blood pressure	≤55 mmHg	Reference	
	>55 mmHg	2.46 (1.52–3.96)	**<0.001**
Mean arterial pressure	≤74 mmHg	Reference	
	>74 mmHg	2.15 (1.32–3.49)	**<0.001**
Surgeon	˂5 year experience	Reference	
	>5 year experience	1.68 (0.99–2.85)	0.056
Time of surgery	≤30 min	Reference	
	>30 min	14.43 (4.97–41.88)	**<0.001**

The bold means statistically significant results.

**Table 3:** Linear regression model.

Predictor	Beta	95% IS for Beta	*P*
Increase of MAP per 1 year of age	0.70	0.26–1.14	0.002
Difference MAP bleeding vs. no bleeding	2.10	0.00–4.25	0.056

**Table 4:** Multidimensional logistic regression model.

Predictor		OR (95% Confidence Interval)	*P*
Age	≤4 years	Reference	
	>4 years	1.21 (0.71–2.06)	0.481
MAP	≤74 mmHg	Reference	
	>74 mmHg	2.19 (1.30–3.69)	**0.003**
Time of surgery	≤30 min	Reference	
	>30 min	13.57 (4.61–39.97)	**<0.001**

The bold means statistically significant results.

## Data Availability

The data presented in this study are available on request from the corresponding author.
